# Detection of vaccine-derived poliovirus type 2 amid the burden of infectious diseases in the UK: A cause for alarm

**DOI:** 10.1016/j.amsu.2022.104773

**Published:** 2022-09-22

**Authors:** Ateeba Kamran, Maira Salman, Rida Siddiqui, Noor Zehra Shabbir, Samia Rohail, Mahnoor Sukaina

**Affiliations:** Karachi Medical and Dental College, Karachi, Pakistan

## Abstract

The re-emergence of polio in the UK reminds us that the global threat of polio remains. Viruses know no bounds or borders. COVID-19, Monkeypox, and polio are evidence of this. Poliomyelitis was once the leading cause of death and paralysis in the UK and globally. With the introduction of vaccines during the 1950s–1960s, polio was eradicated from most developed countries, including the UK. The last case of wild polio was in 1984, and the UK was polio-free in 2003. The recent detection of vaccine-derived poliovirus type 2 in London sewage samples is alarming. Routine inoculations were adversely affected due to COVID-19, and UK's wastewater monitoring program was suspended due to economic restrictions. The UK faces several challenges with the burden of COVID-19, Monkeypox, and the return of polio could further burden the already pandemic-stricken healthcare system. To prevent further epidemics in the UK, wastewater sampling remains crucial in evaluating, searching, and eradicating the spread of poliovirus. Further epidemiological surveillance in adjacent areas to the Beckton plant is crucial to filling any gaps in understanding the outbreak's extent and guiding the initiation of appropriate and timely public health measures. The importance of vaccination in unvaccinated or incompletely vaccinated individuals cannot be overstated, especially in areas where vaccination rates are low. The risk of polio remains globally until its complete eradication from endemic countries. Until elimination, a global effort should be made to minimize the risk and the consequent spread of poliovirus by maintaining strong population immunity levels through high vaccination coverage.

In the wake of the COVID-19 pandemic, the UK has been hit with a barrage of infectious diseases. With the Omicron sub-variants driving the wave of infections, the re-emergence of Monkeypox, occurrences of Lassa fever, and detection of Crimean-Congo hemorrhagic fever, the UK has seen a considerable rise in infectious diseases [[1], [2], [3], [4]]. With this burden of syndemics plaguing the Kingdom, it now faces a new threat. The detection of polio in several sewage samples from the London Beckton Sewage Treatment Works has led to the declaration of a national incident [5]. Many deadly epidemics have struck London over the centuries, and emerging infectious diseases all point to the possibility of further outbreaks [6].

95% of the infected individuals have a subclinical presentation [7]. They are carriers and shed the virus through their feces [7]. 4–8% are mild infections called abortive poliomyelitis [7]. Gastroenteritis and respiratory tract infections are seen with symptoms like headaches, sore throat, fever, nausea, and diarrhea [7]. 1% of the cases present with aseptic meningitis (non-paralytic poliomyelitis), causing fever, malaise, headache, and neck and back stiffness [7]. In less than 1% of individuals, paralytic poliovirus is the gravest manifestation [7]. One in every 200 infections causes flaccid paralysis [8]. 5–10% of cases are fatal because of respiratory compromise [8]. 25–50% of individuals may develop post-polio syndrome 15–40 years after the resolution of initial infection, characterized by muscle weakness and pain [9].

Between February and May, the UK Health Security Agency (UKHSA) repetitively identified poliovirus in the sewage in North and East London [10,11]. This was the vaccine-like type 2 poliovirus (SL2) [10,11]. Prior sewage monitoring discovered 1–3 unrelated SL2 yearly [10,11]. These were always one-time occurrences due to an individual vaccinated with the oral polio vaccine (OPV) overseas, presumably in Afghanistan or Pakistan, leaving traces of SL2 in their feces [10,11]. Towards the end of June, new samples raised concern because the discovered strain seemed to have evolved into a more wild type [10,11]. This suggests that the virus has been mutating as it spread. This is the vaccine-derived poliovirus type 2 (VDPV2) [10,11]. VDPV2's presence suggests the potential to revert to a fully virulent poliovirus. Between 8 February and 5 July, 116 cases were detected from 19 wastewater samples [12]. Further upstream sampling by UKHSA identified the virus in multiple boroughs and adjacent areas in the South and East of Beckton [12].

The general repercussions of COVID-19, including anti-vaccine sentiment, restricted movement, lockdowns, and logistical challenges, affected routine childhood vaccination programs in the UK [13]. The poliovirus detection in London sewage samples has highlighted the utility of wastewater surveillance. The EMHP SARS-CoV-2 wastewater monitoring program was adjourned by March 2022 due to economic constraints [14]. Without close monitoring, the first indication of polio circulating in a population could be a case of paralysis or death [10]. Recurring detection of VDPV2 in London's sewage samples poses a serious threat to the UK and implies its active transmission [15]. Vaccinated people, albeit asymptomatic, can spread the virus through respiratory secretions or the fecal-oral route. Thus, it can spread to low vaccination rate areas and affect unvaccinated or partially vaccinated individuals [15]. According to UKHSA, this is precisely what happened in London. Because unvaccinated people shed the virus for extended periods, their role during outbreaks and epidemics is critical [10].

The World Health Organization (WHO) sets a 95% population target for full immunization by age 5. Only 94% of two-year-olds and 95% of 5 years old in the UK have received a polio vaccine, with London covering only 89% of 2-year-olds and 91.2% of five-year-olds [16,17]. Complete immunization renders an indispensable teenage booster shot. London continues to fall behind. Hillingdon had the lowest coverage nationwide, with 35% of 13 and 14-year-olds vaccinated, while Brent covered 38%. Four local authorities, Westminster, Hammersmith and Fulham, Camden, and Ealing, had lower than 50% coverage [16,17]. Such low proportions among teenagers intensify the risk of infection. The administration of boosters is commissioned under NHS England and Improvement via a school-based delivery model but was disrupted during the lockdown. Despite reopening schools in September 2020, attendance remained unsatisfactory due to ongoing infection and migration of people from local authorities, thus affecting vaccination programs [17]. Overall, London's low vaccination rates could be attributed to public health funding cuts concerning Brexit, mass emigration from the city during Covid-19, diversity of London's population, and impoverishment [15].

To control this outbreak, the UKHSA has already strengthened routine surveillance, investigating wastewater samples in the adjacent areas in contact with the Beckton plant and sampling 8 sites across London [12]. To ascertain whether poliovirus is spreading outside of London, 15 further sites in London and 10–15 nationally will start sewage sampling [12]. The surveillance of wastewater plants occupies a pivotal role in eradication, especially when infections spread silently or when early detection helps give a prior warning [18]. Therefore, a collaboration between researchers, water management, and funding committees is vital. Better investment in such projects is paramount in identifying potential threats and ensuring public health [18]. Monkeypox is evidence of this. It started in 2022 with just 7 confirmed cases on May 6 and has reached 2914 cases by 8 August [2].

Zoonotic diseases such as COVID-19 and Monkeypox are difficult to eliminate through mass vaccination campaigns due to their animal reservoirs [19]. Polio, however, is a viral infection and has a favorable probability. High vaccination rates maintain high levels of population immunity [20]. Since 1988, cases have decreased by over 99%, from 350,000 to only 19 cases reported in 2022, demonstrating the benefits of immunization [8]. The government has commanded individuals to ensure that they and their families are up to date with vaccinations [12]. A booster dose of the inactivated polio vaccine (IPV) would be given to every child between the ages of 1 and 9 in London due to its comparatively low vaccination coverage [12]. The NHS will contact the parents when it is time for the childhood vaccination catch-up or booster dose [12]. Accepting this dose is crucial to bolstering protection even if vaccinations are up to date. The impacted areas with low vaccination rates will be the program's first stop [21]. Parents are urged to contact UKHSA and seek medical care if they experience muscle weakness or suspect acute flaccid paralysis (AFP). Individuals with AFP should be examined for poliovirus by taking throat swabs and two stool samples 48 hours apart [22].

Viruses cannot be confined by borders. The UK was declared polio-free in 2003 [23]. This recurrence comes after almost 40 years, highlighting the need for polio vaccination worldwide [23]. Necessary steps should be undertaken to reach vulnerable populations to improve mass immunization and surveillance to halt the string of infectious diseases plaguing the UK [24]. The risk of polio remains globally as long as even a single individual remains unvaccinated or infected [24]. Polio detection in Israel and a case of paralysis in New York for the first time in several years is a wake-up call [25]. The UKHSA is working with the WHO and health specialists in the US and Israel to examine the links between polioviruses identified there [26]. Genetic analyses revealed vaccine-derived viruses in the three countries [27]. Since 2017, there have been 396 cases caused by the wild poliovirus versus more than 2600 by OPV [27]. 115 out of 127 cases have been attributed to VDPV2 in northern Yemen, northern Nigeria, and eastern DR Congo in 2022 [28]. The spillover between Nigeria and West Africa highlights the threat of international spread [28]. Wild poliovirus is still endemic in Pakistan and Afghanistan [29]. Vaccination campaigns encounter challenges in these historically fragile healthcare systems, including vaccine hesitancy, inadequate health infrastructure, and political unrest [30]. Due to its easy administration and cheaper cost, OPV remains a keyhole in eliminating polio from endemic countries [31]. The Americas, Europe, and Africa accomplished elimination solely through OPV [31]. However, the emergence of vaccine-derived poliovirus (VPDV) drove many countries, including the UK, to switch toward IPV [32]. In 2020, the WHO approved the novel oral polio vaccine type-2 [29]. It is genetically stable and has no hazard of VPDV [33]. It is being utilized for mass inoculation campaigns, and with supply increments, it would replace OPV [29]. Support should be provided to low-income nations to access vaccines, strengthen surveillance, and enable easy accessibility for front-line health workers to ease vaccine delivery [30]. Timely intervention can prevent a national and subsequent global health catastrophe.

## Ethical approval

N/A.

## Sources of funding

None.

## Author contribution

Ateeba Kamran conceived the idea and did the submission. Ateeba Kamran, Maira Salman, Rida Siddiqui, Noor Zehra Shabbir, and Samia Rohail retrieved the data and made the first draft. Ateeba Kamran updated the manuscript and edited the final draft. Mahnoor Sukaina proofread the final draft. All authors have critically reviewed and approved the final draft.

## Registration of research studies

1. Name of the registry: N/A.
2. Unique Identifying number or registration ID: N/A.
3. Hyperlink to your specific registration (must be publicly accessible and will be checked): N/A.

## Guarantor

N/A.

## Consent

N/A.

## Declaration of competing interest

None.
